# Factors influencing healthcare worker symptomatic respiratory infection and vaccine uptake during the post-COVID-19 pandemic period

**DOI:** 10.1017/ash.2025.10094

**Published:** 2025-08-13

**Authors:** Liam Townsend, Lisa Domegan, Wenzhou Wang, Siobhan Quirke, PRECISE Steering Group, Colm Bergin, Catherine Fleming

**Affiliations:** 1 Department of Infectious Diseases, St James’s Hospital, Dublin, Ireland; 2 Department of Clinical Medicine, Trinity College Dublin, Dublin, Ireland; 3 Health Protection Surveillance Centre Dublin, Dublin, Ireland; 4 Royal College of Surgeons Ireland, Dublin, Ireland; 5 Department of Infectious Diseases, University Hospital Galway, Galway, Ireland; 6 School of Medicine, University of Galway, Galway, Ireland

## Abstract

**Objective:**

Investigate the factors associated with symptomatic respiratory infection and uptake of seasonal SARS-CoV-2 and influenza vaccine amongst healthcare workers (HCWs).

**Design:**

Longitudinal prospective multi-center study.

**Setting:**

Two tertiary healthcare centers in Ireland.

**Participants:**

N = 893 self-selected HCWs across all disciplines.

**Methods:**

Monthly self-reported questionnaires from September 2024 to February 2025 completed by all participants, providing infection symptoms, self-testing for COVID-19 and receipt of vaccination against SARS-CoV-2 or influenza in the preceding 30 days. Additional data collected included comorbidities, known diagnosis of Long COVID, demographic data, prior infection and vaccination status, and healthcare role. Multivariable logistic regression models assessed the factors associated with symptom development, self-testing, and vaccine uptake.

**Results:**

Symptomatic respiratory illness was reported by n = 321 (36%) of participants during the study period, with a preexisting diagnosis of Long COVID associated with developing symptoms. Testing for COVID-19 was performed by 63% (n = 202) of symptomatic individuals, with a shorter duration since prior infection the only significant predictor of self-testing. Vaccine uptake was variable, with 37% receiving influenza and 22% receiving SARS-CoV-2 vaccination for that period. Older age and shorter interval since previous vaccine were associated with increased uptake of both vaccines, while men were more likely to be vaccinated against COVID-19.

**Conclusions:**

In the postpandemic period, self-reported symptomatic respiratory infections remain common amongst HCWs. The legacy of the pandemic influences this, with a preexisting diagnosis of Long COVID associated with increased symptom burden, while low vaccination rates and understanding the factors associated with this present a challenge to ongoing risk mitigation.

## Introduction

The COVID-19 pandemic had a large impact on healthcare systems worldwide and disproportionately affected healthcare workers (HCWs). This lead to reconfiguration of normal workplace practices and the widespread use of personal protective equipment.^1,2^ The impact on healthcare systems was compounded by HCW illness and associated workdays lost.^3^ As the pandemic evolved, the burden of acute COVID-19 on healthcare systems and HCWs lessened, and a gradual return to prepandemic workplace practices took place. HCWs still remain at increased risk of communicable infections, particularly viral upper respiratory tract infections.^4^ In addition to the risks these infections pose to the infected HCWs, there are also associated risks of nosocomial outbreaks amongst vulnerable patient groups.^5^

HCW respiratory illness has been mitigated by the availability of seasonal influenza vaccination and, more recently, COVID-19 booster vaccines. However, vaccine uptake amongst HCWs is highly variable, with reduced vaccine uptake seen in younger, female, and non-clinical HCWs.^6,7^ This is particularly relevant for COVID-19 booster vaccination, as public perception toward COVID-19 vaccination has evolved over the course of the pandemic and vaccine hesitancy is a growing issue.^8^ In this postpandemic period, healthcare systems continue to be challenged by circulating viral infections on a seasonal basis. The Prevalence of Antibodies to SARS-CoV-2 and other Viral Infections in Irish HCWs (PRECISE Plus) study is a multicentre longitudinal study of viral respiratory tract infections, vaccine uptake and immune responses in HCWs.^9–11^ We utilized this cohort to evaluate the frequency and predictors of symptomatic upper respiratory infections in HCWs during the six-month period from September 2024 to February 2025, capturing the seasonal winter surge. We also evaluated the factors associated with undertaking self-taken point of care COVID-19 tests, and factors associated with uptake of both seasonal influenza and COVID-19 booster vaccination. These results will be used to inform practice and policy for prevention and early detection of transmissible infections in HCWs.

## Methods

### Study setting and participants

This was a multisite prospective study at two hospital sites in Ireland: St James’s Hospital (SJH), Dublin, and University Hospital Galway (UHG). These sites were chosen at the start of the COVID-19 pandemic due to the contrasting COVID-19 seroprevalence in their catchment areas.^12^ All HCWs, defined as any person employed to work at the hospital sites, were invited to take part. Vaccination was conducted on-site for both SARS-CoV-2 and seasonal influenza, and was promoted via hospital communications and peer leaders.

Irish HCWs were not subjected to a COVID-19 vaccine mandate during the pandemic, nor were they obliged to disclose their vaccination status to their employer. There has also never been an influenza vaccine mandate for HCWs in Ireland. There were wider societal restrictions on citizens who did not receive COVID-19 vaccination, such as limitations on entry to pubs, restaurants and cafes.

Ethical approval for the study was obtained from the local ethics committees at SJH and UHG (application No. TUH/SJH REC 2022—Nov—23002300 and GCREC 15/09/2022 C.A. 2860, respectively). Electronic informed consent was obtained from all participants.

### Data collection and variables

Data collection was paperless, with participants providing electronic consent at time of enrollment. Electronic questionnaires were emailed to participants every month, in addition to an enrollment questionnaire. The enrollment questionnaire collected demographic data including age, sex, ethnicity, education level and HCW role, as well as medical history and prior COVID-19 infection status. HCW role was further characterized as clinical or non-clinical. Participants were specifically asked if they had a preexisting diagnosis of Long COVID, which was defined as any current or previous unexplained symptom following SARS-CoV-2 infection lasting more than 3 months.^13^ Monthly questionnaires recorded episodes of symptomatic illness during the preceding month, and duration of these symptoms. Number of tests conducted for COVID-19 and influenza and their results in the preceding month were self-reported by participants, as were the indications for each test and the type of test used (antigen test or polymerase chain reaction (PCR) test). Point of care tests were available at both workplaces to HCWs, free of charge. Receipt of COVID-19 and influenza vaccines in the preceding month was also self-reported. Vaccine records were confirmed by cross-referencing with COVAX, the Irish national immunization system, which is a mandatory reporting system and records all immunizations received by individuals in Ireland. Participants who did not complete their monthly questionnaire received a reminder email after two weeks.

### Statistical analysis

Descriptive statistics are reported as means with standard deviations (SD) and medians with interquartile ranges (IQR), as appropriate. Univariate analysis was used to analyze differences between HCWs who had incident infection and those who did not, as well as those who received vaccination and those who did not. Multivariable linear regression was used to analyze factors associated with incident infection and vaccine uptake. Variables known to be associated with the outcomes (age and sex) were chosen for inclusion *a priori*, while any significant variables on univariate analysis were also included. Any HCW role that represented <1% of the total cohort were grouped together for analysis. Statistical significance was considered at the .05 level. All analysis was performed using Stata version 18.0 (Stata Statistical Software).

## Results

Data was collected from n = 893 HCWs over the study period, with n = 584 (65%) from SJH and n = 309 from UHG, representing 11% of all HCWs across both sites. The average response rate per month was 73%, with all participants providing responses for a minimum of four of the study months. SJH participants were younger than those in UHG, while UHG participants were more likely to co-habit with another HCW and had higher educational attainment. Ethnicity, sex, comorbidities and seasonal vaccine uptake were similar across both sites. Long COVID was reported by n = 36 (4%) of the cohort. All participants received a primary COVID-19 vaccine course; n = 228 (26%) received one booster, n = 197 (22%) received two boosters, and 315 (35%) received three booster doses. A complete description of the cohort is shown in Table 1.

**Table 1.** Cohort characteristics by study site

	Total cohort n = 893	SJH n = 584	UHG n = 309	
Age, years; median (IQR)	45 (37–53)	44 (35–53)	48 (40–55)	z = −4.30, *P* < .0001
Sex, female; n (%)	720 (81)	460 (79)	260 (84)	χ^2^ = 3.74, *P* = .053
Ethnicity; n (%) White Irish Asian/Chinese Asian/Other Black/African White/Other Other No data	735 (82) 11 (1) 41 (5) 9 (1) 76 (9) 8 (<1) 13 (1)	483 (83) 6 (1) 32 (5) 7 (1) 44 (8) 5 (<1) 7 (1)	252 (82) 5 (2) 9 (3) 2 (<1) 32 (10) 3 (<1) 6 (2)	r^2^ = .01, *P* = .34
Education level; n (%) Secondary Third level Post graduate	108 (12) 379 (42) 406 (45)	85 (15) 246 (42) 253 (43)	23 (7) 133 (43) 153 (50)	r^2^ = .01, *P* = .01
Clinical, yes; n (%)	488 (55)	317 (54)	171 (55)	χ^2^ = .21, *P* = .65
Role; n (%) Nursing Doctor HCA AHP Administration Catering Laboratory Technician Other	268 (30) 123 (14) 38 (4) 122 (14) 174 (19) 12 (1) 57 (6) 11 (1) 88 (10)	161 (28) 82 (14) 28 (5) 77 (13) 128 (22) 11 (2) 36 (6) 5 (<1) 56 (10)	107 (35) 41 (13) 10 (3) 45 (15) 46 (15) 1 (<1) 21 (7) 6 (2) 32 (10)	r^2^ = .02, *P* = .04
Underlying risk factor, yes; n (%)	112 (13)	80 (14)	32 (10)	χ^2^ = 1.51, *P* = .22
Live with HCWs; yes (%)	136 (15)	85 (15)	51 (17)	χ^2^ = .0003, *P* = .99
Time since last COVID vaccine, days; median (IQR)	726 (481–1 029)	724 (335–1 026)	835 (584–1 029)	z = −1.52, *P* = .13
COVID booster uptake; n (%)	199 (22)	129 (22)	70 (23)	χ^2^ = .04, *P* = .85
Influenza vaccine uptake; n (%)	334 (37)	209 (36)	125 (40)	χ^2^ = 1.88, *P* = .17

Between-group differences assessed using χ^2^, Wilcoxon rank-sum and ANOVA, as appropriate. SJH, St James’s Hospital; UHG, University Hospital Galway; HCA, healthcare assistant. AHP, Allied Healthcare Professional.

### Symptomatic illness and factors associated with same

The majority of participants (n = 681, 76%) reported COVID-19 infection prior to September 2024. The total number of prior infections reported was 1,107 (median 1, IQR 1–2), with median time from last confirmed COVID-19 infection to enrollment of 366 days (IQR 122–615). Testing practices changed over time, with the majority of index infections being diagnosed via PCR test, while the majority of subsequent infections were diagnosed by antigen testing.

**Figure 1.**
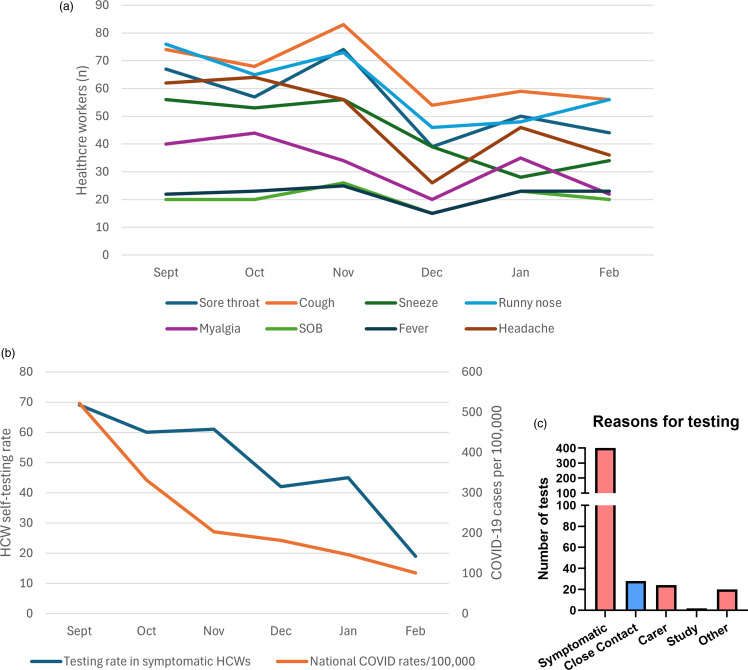
Frequency and variety of symptoms reported during study period (A), testing rates among symptomatic healthcare workers (HCWs) in the context of national SARS-CoV-2 infection rates (B), and reasons provided for undertaking point-of-care SARS-CoV-2 antigen testing (C).

There were 574 episodes of symptomatic illness during the six-month study period, reported by n = 321 (36%) participants. The most common symptoms reported were sore throat, cough, runny nose, and headache (Figure 1A). The median number of days spent symptomatic was 5 (IQR 4–7).

Symptomatic illness was associated with having a prior COVID infection and having a preexisting diagnosis of Long COVID. Higher educational attainment was also associated with increased likelihood of developing a symptomatic case, however receipt of COVID-19 booster within the preceding year and time since most recent COVID-19 infection were not associated with symptom development (Table 2). The factors associated with developing upper respiratory tract infection symptoms were further investigated using a multivariable logistic regression model, including the significant univariate variables as well as those identified *a priori*, namely age, receipt of COVID-19 vaccine in the preceding year, and being in a clinical role. Having a diagnosis of Long COVID remained significantly associated with the development of viral URTI symptoms (Table 3).

**Table 2.** Comparison between HCWs with a symptomatic case versus those without

	Symptomatic (n = 321)	No symptoms (n = 572)	
Site, SJH; n (%)	206 (64)	378 (66)	χ^2^ = .33, *P* = .57
Age	45 (37–53)	46 (37–54)	z = .75, *P* = .46
Sex, female; n (%)	259 (81)	464 (81)	χ^2^ = .001, *P* = .97
Education level Secondary Third level Post graduate	29 (9) 120 (37) 172 (54)	79 (14) 259 (45) 234 (41)	r^2^ = .02, *P* = .001
Known risk factor; n (%)	35 (11)	77 (13)	χ^2^ = 2.42, *P* = .12
Live with HCW, yes	63 (20)	73 (13)	χ^2^ = .19, *P* = .67
Clinical role, yes; n (%)	178 (55)	310 (54)	χ^2^ = .01, *P* = .91
Long COVID, yes; n (%)	22 (7)	14 (2)	χ^2^ = 10.32, *P* = .001
COVID booster/flu vaccine prior to symptoms, yes; n (%)	110 (34)	172 (30)	χ^2^ = 1.63, *P* = .20
Previous COVID infection, yes; n (%)	271 (84)	410 (72)	χ^2^ = 6.77, *P* = .009
Time since last COVID infection, days; median (IQR)	387 (121–624)	355 (127–598)	z = -.86, *P* = .39
Time since last COVID vaccine, days; median (IQR)	720 (584–1 028)	729 (334–1 037)	z = .45, *P* = .66
COVID boosters in last 365 days, yes; n (%)	56 (17)	116 (20)	χ^2^ = 1.53, *P* = .22

Between-group differences assessed using χ^2^, Wilcoxon rank-sum and ANOVA, as appropriate.

**Table 3.** Multivariable logistic regression model to identify features associated with symptomatic illness and self-testing

Factors associated with symptomatic disease		
	Odds ratio (95% CI)	P value
Education level Secondary Third level Postgraduate	.63 (.35–1.12) Reference 1.41 (.99–1.98)	.11 Reference .051
Age	.99 (.98–1.01)	.29
COVID-19 booster in last 365 days	.72 (.50–1.04)	.08
Clinical role	.79 (.55–1.10)	.15
Previous COVID infection	1.53 (1.98–2.41)	.06
Prior diagnosis of Long COVID	2.62 (1.22–5.64)	.01
Factors associated with self-testing for COVID-19 in symptomatic HCWs		
	Odds ratio (95% CI)	P value
Sex, male	.58 (.27–1.25)	.16
Preexisting factor	2.49 (.79–7.92)	.12
Time since last infection	.70 (.51–.96)	.03

Multivariable logistic regression, with all variables included in the model shown in the table. Time since last vaccine was z-scored prior to analysis.

### Self-testing for COVID-19 in symptomatic HCWs

Of the n = 321 individuals with symptoms, there were 474 self-administered antigen tests for COVID-19 performed by n = 202 (63%) participants. Testing frequency was in line with national rates of COVID-19 cases (Figure 1B). The majority of tests performed (n = 400, 84%) were due to symptomatic illness, while other indications for testing included being a close contact of a known case and being in contact with vulnerable individuals (Figure 1C). The only factor associated with performing more than one COVID-19 test during the study period was having a preexisting risk factor for COVID-19 acquisition.

Factors associated with self-testing were investigated. Self-testing was associated with female sex, those with a preexisting risk factor for COVID-19, and those with a shorter interval since prior infection (Supplemental Table 1). Following adjustment in a multivariable logistic regression model, only shorter duration since last confirmed COVID-19 infection was associated with undertaking a self-taken COVID-19 test (Table 3).

There were 34 positive COVID-19 results, representing 17% (34/202) of the population who undertook a test and 4% (34/893) of the total study population. There were no variables identified with an increased likelihood of having a positive COVID-19 test, with no relationship between incident COVID-19 infection with time since most recent infection or time since last vaccination (Supplemental Table 2).

### Uptake of seasonal influenza and SARS-CoV-2 booster vaccination

**Figure 2.**
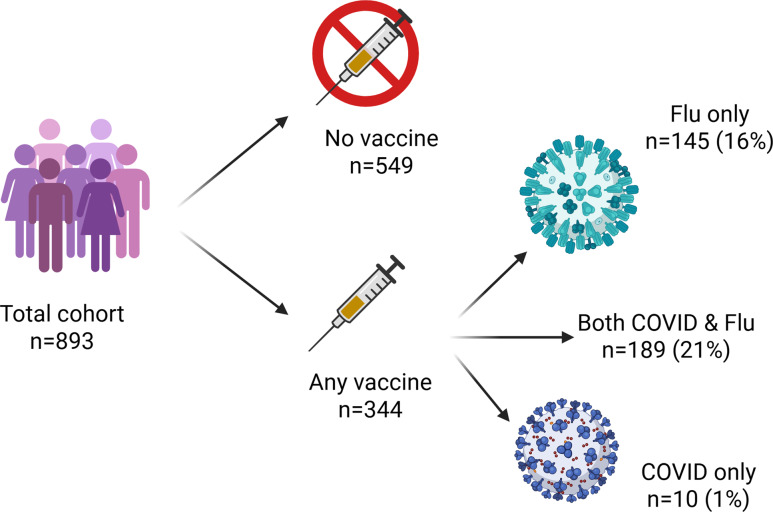
Uptake of influenza and SARS-CoV-2 vaccines.

All participants had access to the seasonal influenza and SARS-CoV-2 booster vaccines in their workplace. N = 344 (38%) received at least one booster vaccine; n = 145 (16%) received the influenza vaccine alone, n = 10 (1%) received the SARS-CoV-2 booster alone, and n = 189 (21%) received both (Figure 2). HCWs who received any vaccine during the winter period were older, had higher educational attainment, and had a shorter time since their prior vaccine compared to those who did not receive a seasonal vaccine. There were no differences in vaccine uptake comparing clinical and non-clinical HCWs, but Allied Health Professionals were more likely to have received a vaccine. Increasing age, shorter interval since last vaccination, and being an Allied Health Professional remained associated with increased vaccine uptake following multivariable logistic regression.

The associations with each individual vaccine were then explored. Similar to receipt of any vaccine, increased age, higher educational attainment, and shorter interval since most recent vaccination were all associated with receipt of both COVID-19 and influenza vaccination (Table 4). However, males were significantly more likely to receive a COVID-19 vaccine, while no sex differences were seen in influenza vaccination uptake. Following multivariable regression, age, sex, and time since last vaccination were all significantly associated with COVID-19 vaccine receipt, while age and time since last vaccination were associated with influenza vaccine uptake (Table 5). The sex differences seen in COVID-19 vaccine uptake were investigated further. Females aged < 50 years of age had significantly lower uptake than females older than 50 years of age (*P* = .01), while females older than 50 had similar rates of vaccine uptake to men (*P* = .44).

**Table 4.** Factors associated with uptake of SARS-CoV-2 booster and seasonal influenza vaccine

	SARS-CoV-2 booster			Seasonal Influenza vaccine		
	No booster n = 694	Booster n = 199		No vaccine n = 559	Vaccine n = 334	
Site, SJH; n (%)	455 (66)	129 (65)	χ^2^ = .04, *P* = .85	375 (67)	209 (63)	χ^2^ = 1.88, *P* = .17
Age	44 (36–52)	49 (41–56)	z = −4.99, *P* < .0001	44 (35–51)	48 (40–56)	z = −5.57, *P* < .0001
Sex, female; n (%)	571 (82)	149 (75)	χ^2^ = 5.43, *P* = .02	452 (79)	268 (80)	χ^2^ = .05, *P* = .82
Education Secondary Third level Postgraduate	92 (13) 310 (45) 292 (42)	16 (8) 69 (35) 114 (57)	r^2^ = .02, *P* = .001	76 (14) 254 (45) 229 (41)	32 (10) 125 (37) 177 (53)	r^2^ = .01, *P* = .002
Clinical role, yes; n (%)	374 (54)	114 (57)	χ^2^ = .45, *P* = .50	296 (53)	192 (57)	χ^2^ = 1.30, *P* = .25
Role; n (%) Nursing Doctor HCA AHP Administration Catering Laboratory Technician Other	216 (31) 81 (12) 34 (5) 87 (13) 147 (21) 11 (2) 36 (5) 10 (1) 72 (10)	52 (26) 42 (21) 4 (2) 35 (18) 27 (14) 1 (<1) 21 (11) 1 (<1) 16 (8)	r^2^ = .04, *P* = .0001	170 (30) 68 (12) 28 (5) 60 (11) 124 (22) 10 (2) 32 (6) 9 (2) 58 (10)	98 (29) 55 (16) 10 (3) 62 (19) 50 (15) 2 (<1) 25 (7) 2 (<1) 30 (9)	r^2^ = .03, *P* = .001
Risk factor, yes; n (%)	87 (13)	25 (13)	χ^2^ = .13, *P* = .72	69 (12)	43 (13)	χ^2^ = .02, *P* = .89
Previous COVID infection	522 (75)	159 (80)	χ^2^ = .36, *P* = .55	432 (77)	265 (79)	χ^2^ = .11, *P* = .74
Time since last COVID infection	353 (125–616)	404 (115–614)	z = −.45, *P* = .65	353 (128–631)	387 (121–588)	z = .10, *P* = .92
Time since last vaccine, days; median (IQR)	898 (612–1 096)	674 (333–737)	z = 5.10, *P* < .0001	946 (612–1 171)	682 (334–946)	z = 4.90, *P* < .0001

Between-group differences assessed using χ^2^, Wilcoxon rank-sum and ANOVA, as appropriate. HCA, healthcare assistant; AHP, Allied Healthcare Professional.

**Table 5.** Multivariable logistic regression model to identify features associated with SARS-CoV-2 booster and seasonal influenza vaccination

	SARS-CoV-2 booster		Seasonal Influenza	
	Odds ratio (95% CI)	P value	Odds ratio (95% CI)	P value
Age	1.05 (1.03–1.07)	<.0001	1.05 (1.03–1.07)	<.0001
Education level Secondary Third level Postgraduate	.79 (.37–1.70) Reference 1.05 (.68–1.62)	.55 Reference .83	.80 (.42–1.51) Reference 1.22 (.84–1.78)	.49 Reference .30
Time since last vaccine	.64 (.52–.77)	<.0001	.70 (.60–.83)	<.0001
Sex, male	1.83 (1.12–2.97)	.02	1.07 (.68–1.68)	.76
Role; n (%) Nursing Doctor HCA AHP Administration Catering Laboratory Technician Other	Reference 1.79 (.98–3.26) .66 (.20–2.16) 1.55 (.86–2.78) .65 (.35–1.21) .61 (.05–6.92) 2.29 (1.10–4.77) – .76 (.33–1.74)	Reference .06 .49 .14 .17 .69 .03 – .52	Reference 1.48 (.87–2.54) .70 (.27–1.84) 2.13 (1.26–3.58) .70 (.41–1.18) .82 (.11–5.84) 1.34 (.68–2.66) .29 (.03–2.88) 1.04 (.52–2.04)	Reference .15 .47 .005 .18 .84 .40 .29 .92

All variables shown were included in the model. Time since last vaccine was z scored prior to analysis. HCA, healthcare assistant; AHP, Allied Healthcare Professional.

## Discussion

HCWs are at increased risk of transmissible infections compared to the general population.^14,15^ While in recent years SARS-CoV-2 has been the pathogen of concern in all populations, as reflected by more than three-quarters of our study population reporting prior COVID-19 infection, other seasonal respiratory viruses remain a significant threat to HCW health. This study demonstrates the high burden of such infections, with 36% of participants experiencing symptoms of viral respiratory tract infection from September 2024 to February 2025. The approach to testing by symptomatic HCWs is variable, with one-third not undertaking any point of care test. The rate of positive SARS-CoV-2 tests in symptomatic individuals was 17%, demonstrating the burden of disease in HCWs caused by pathogens other than SARS-CoV-2. It may also reflect the reduced sensitivity of antigen tests compared to PCR. Vaccination is an important tool in the prevention of both COVID-19 and influenza infection in HCWs. However, vaccination rates were low, with 37% availing of seasonal influenza vaccine and only 22% receiving a COVID-19 booster. We identify several important factors associated with symptomatic disease and vaccine uptake that should prospectively inform risk reduction strategies in HCWs to prevent clinical disease and onward nosocomial transmission.

Symptomatic illness was more likely in individuals with a preexisting diagnosis of Long COVID. The role of Long COVID is striking. Despite only representing 4% of the total cohort, it is the strongest predictor of developing symptomatic upper respiratory tract infection. The pathogenesis of Long COVID remains poorly-understood, with several possible disease pathways proposed.^16,17^ However, investigations of ongoing immune defects predisposing to recurrent infections have not demonstrated any conclusive proof of increased infection susceptibility.^18,19^ Given the lack of a biological cause for Long COVID to be associated with symptomatic episodes, it may reflect differing HCW behaviors. There is a well-established culture of presenteeism among HCWs despite ill-health, leading to under-reporting of symptoms.^20,21^ In contrast, individuals with Long COVID have increased utilization of healthcare resources, in particular ambulatory care.^22,23^ There is growing evidence that recurrent COVID-19 infection, rather than Long COVID, may increase susceptibility to repeated COVID-19 infection.^24^ This emphasizes the importance of infection prevention. We also observed monthly heterogeneity in the frequency of symptom episodes, with November having the highest rate of symptomatic illness. National surveillance data for that period suggests that COVID-19 was the most common circulating viral pathogen at that time, with the influenza surge commencing in two weeks later.^25^ This may reflect HCWs acting as disease sentinels of the impending influenza peak.

The influence of health behaviors on self-reporting symptoms likely also influences self-testing for COVID-19. There are significant costs associated with HCW absences due to infection, and presenteeism may influence decisions to test.^26^ A shorter time since most recent infection was the only significant predictor of undertaking a point of care antigen test for COVID-19. The use of self-taken antigen tests is in line with national guidance for HCWs, with closure of on-site PCR testing facilities for staff.^27^ Point of care tests are not provided in the workplace, with the onus on the individual HCW to source a test kit. Additionally, point of care antigen tests are less sensitive than viral PCR tests, which may result in false-negative tests.^28,29^

There were low rates of vaccine uptake in our cohort, in particular against SARS-CoV-2, with less than a quarter of HCWs receiving a COVID-19 booster. Our influenza vaccine uptake rate of 37% is markedly lower than the national prepandemic vaccination rate of 53%.^30^ Older age and shorter interval since most recent vaccination were associated with increased uptake of both COVID-19 and influenza vaccination. Age is well-recognized as a factor associated with vaccine uptake, even in cohorts such as ours, where most participants are below the threshold for age-based vaccine recommendations.^31^ A shorter time interval since previous vaccine likely again reflects health behaviors, with individuals who have previously been engaged in a vaccine program more likely to accept subsequent vaccines.^32^ Interestingly, males were more likely to receive the COVID-19 booster, whereas there were no sex differences in influenza vaccine uptake. Concerns regarding reproductive health have previously been associated with reduced SARS-CoV-2 vaccination in females, despite large studies demonstrating no increased rates of adverse outcomes.^33,34^ Our data would support the ongoing legacy of these concerns, with the sex differences only seen in premenopausal (<50 yr of age) HCWs. It is also notable that laboratory staff have significantly higher COVID-19 vaccine uptake than nurses. Nurses and healthcare assistants had the highest rates of COVID-19 seropositivity early in the pandemic due to their high patient-facing time.^35^ The low COVID-19 vaccine rate is particularly concerning given the association we observe between Long COVID and symptomatic viral infections. Receipt of COVID-19 vaccine is linked with reduced rates of Long COVID, and can therefore be used as a tool to not only prevent acute infection but to mitigate the risk of postinfectious ill-health, recurrent symptomatic episodes, and missed workdays.^36–38^ Concomitant administration of influenza and SARS-CoV-2 vaccines appear efficacious, and there are multicomponent vaccines in development.^39,40^ However, our observed significant differences in vaccine uptake against these pathogens suggest that vaccine education and promotion would be required to mitigate a decline in uptake.

There are several limitations to this study worth noting. Symptoms and tests were self-reported, and are open to recall bias. Given the study design, the cohort may not be wholly representative of the overall HCW population, with 11% of the total eligible HCW population included. However, there is representation across all HCW disciplines in this study. The absence of point of care tests for infections other than COVID-19 prevented us from capturing infections due to influenza, Respiratory Syncytial Virus, and other seasonal respiratory pathogens. We were unable to identify reported COVID-19 infections were acquired within the workplace or in the community.

This study characterizes the burden of symptomatic upper respiratory tract infections in HCWs during the winter season, and identifies predictors of same, namely a preexisting diagnosis of Long COVID. This is a novel finding. We note the highest burden of symptomatic disease in HCWs is within two weeks of influenza becoming the dominant respiratory viral pathogen in the general population. This may provide a rationale to perform multiplex respiratory viral screening on HCWs during the winter periods to identify impending disease surges. We also characterize self-testing behavior, as well as factors associated with vaccine uptake. We identify young female HCWs as a cohort with low COVID-19 vaccine uptake, which is not seen with influenza vaccine uptake. These findings should inform vaccine strategies for HCWs, particularly given the development of combined SARS-CoV-2 and influenza vaccines. Collectively, our self-testing data, vaccine rates and the association with symptomatic disease and Long COVID highlight the need to study health behaviors in HCWs, and the influence this has on HCW attitudes toward respiratory infections.

## Supporting information

10.1017/ash.2025.10094.sm001 Townsend et al. supplementary material Townsend et al. supplementary material

## Supplementary material

The supplementary material for this article can be found at https://doi.org/10.1017/ash.2025.10094
