# Supplementary material for: Factors influencing healthcare worker symptomatic respiratory infection and vaccine uptake during the post-COVID-19 pandemic period
Source: Antimicrob Steward Healthc Epidemiol. 2025 Aug 13;5(1):e183. doi: 10.1017/ash.2025.10094 (PMC12345059; doi:10.1017/ash.2025.10094)
Supplement: Townsend et al. supplementary material [file S2732494X25100946sup001.docx]

*Supplemental Table 1: Factors associated with self-testing for COVID-19 in symptomatic HCWs*

|  | No test (n=119) | Test (n=202) |  |
| --- | --- | --- | --- |
| Site, SJH; n (%) | 72 (60) | 134 (66) | χ^2^=1.11, p=0.29 |
| Age, years; median (IQR) | 45 (36 – 54) | 45 (37 – 53) | z=-0.01, p=0.99 |
| Sex, male; n (%) | 30 (25) | 32 (16) | χ^2^=4.22, p=0.04 |
| Education level   - Secondary - Third level - Postgraduate | 9 (8)  47 (39)  63 (53) | 20 (10)  73 (36)  109 (54) | r^2^=0.002, p=0.71 |
| Clinical role, yes; n (%) | 67 (56) | 111 (55) | χ^2^=0.06, p=0.81 |
| Role; n (%)  - Nursing  - Doctor  - HCA  - AHP  - Administration  - Catering  - Laboratory  - Technician  - Other | 30 (25)  23 (19)  6 (5)  16 (13)  20 (17)  1 (<1)  9 (8)  2 (2)  12 (10) | 61 (30)  25 (12)  7 (3)  37 (18)  37 (18) 1 (<1) 11 (5)  2 (<1)  21 (20) | r^2^=0.02, p=0.69 |
| Pre-existing risk factor; n (%) | 7 (6) | 28 (14) | χ^2^=4.53, p=0.03 |
| Live with HCW; n (%) | 24 (20) | 39 (19) | χ^2^=0.11, p=0.74 |
| Receive booster; n (%) | 62 (52) | 104 (51) | χ^2^=0.01, p=0.92 |
| Time since last vaccine, days; median (IQR) | 753 (336 – 1028) | 712 (605 – 1027) | z=0.90, p=0.37 |
| COVID-19 vaccine the preceding 365 days, yes; n (%) | 19 (16) | 37 (18) | χ^2^=0.29, p=0.59 |
| Previous COVID infection; n (%) | 95 (80) | 176 (87) | χ^2^=2.18, p=0.14 |
| Time since last infection, days; median (IQR) | 556 (250 – 653) | 331 (100 – 610) | z=2.21, p=0.03 |

Between-group differences assessed using Chi-squared, Wilcoxon rank-sum and ANOVA, as appropriate. HCA=healthcare assistant. AHP=Allied Healthcare Professional

*Supplemental Table 2: Associations with positive COVID-19 tests in those undertaking self-testing*

|  | No infection  (n=859) | COVID infection (n=34) |  |
| --- | --- | --- | --- |
| Site, SJH; n (%) | 563 (66) | 21 (62) | χ^2^=0.21, p=0.65 |
| Age, years; median (IQR) | 46 (38 – 50) | 45 (37 – 53) | z=-0.10, p=0.92 |
| Sex, female; n (%) | 690 (80) | 30 (88) | χ^2^=1.36, p=0.24 |
| Education level   - Secondary - Third level - Postgraduate | 105 (12)  367 (43)  387 (45) | 3 (9)  12 (35)  19 (56) | r^2^=0.002, p=0.45 |
| Clinical role, yes; n (%) | 470 (55) | 18 (53) | χ^2^=0.07, p=0.79 |
| Pre-existing risk factor; n (%) | 109 (13) | 3 (9) | χ^2^=0.47, p=0.49 |
| Previous COVID vaccine | 676 (79) | 30 (88) | χ^2^=1.25, p=0.26 |
| Previous COVID infection | 653 (76) | 28 (82) | χ^2^=0.41, p=0.52 |
| Time since last infection | 373 (127 -616) | 202 (78 – 615) | z=0.97, p=0.33 |
| Time since last COVID vaccine, days; median (IQR) | 725 (476 – 1029) | 845 (672 – 1238) | z=-1.07, p=0.29 |

Factors associated with a positive COVID-19 test at any point during the study period. Between-group differences assessed using Chi-squared, Wilcoxon rank-sum and ANOVA, as appropriate
